# Vertical transmission of honey bee viruses in a Belgian queen breeding program

**DOI:** 10.1186/s12917-015-0386-9

**Published:** 2015-03-14

**Authors:** Jorgen Ravoet, Lina De Smet, Tom Wenseleers, Dirk C de Graaf

**Affiliations:** Laboratory of Molecular Entomology and Bee Pathology, Ghent University, Krijgslaan 281 S2, B-9000 Ghent, Belgium; Laboratory of Socioecology and Social Evolution, KU Leuven, Naamsestraat 59, B-3000 Leuven, Belgium

**Keywords:** Honey bee, Eggs, Viruses, Negative-strand detection, Vertical transmission

## Abstract

**Background:**

The Member States of European Union are encouraged to improve the general conditions for the production and marketing of apicultural products. In Belgium, programmes on the restocking of honey bee hives have run for many years. Overall, the success ratio of this queen breeding programme has been only around 50%. To tackle this low efficacy, we organized sanitary controls of the breeding queens in 2012 and 2014.

**Results:**

We found a high quantity of viruses, with more than 75% of the egg samples being infected with at least one virus. The most abundant viruses were Deformed Wing Virus and Sacbrood Virus (≥40%), although Lake Sinai Virus and Acute Bee Paralysis Virus were also occasionally detected (between 10-30%). In addition, Aphid Lethal Paralysis Virus strain Brookings, Black Queen Cell Virus, Chronic Bee Paralysis Virus and *Varroa destructor* Macula-like Virus occurred at very low prevalences (≤5%). Remarkably, we found *Apis mellifera carnica* bees to be less infected with Deformed Wing Virus than Buckfast bees (p < 0.01), and also found them to have a lower average total number of infecting viruses (p < 0.001). This is a significant finding, given that Deformed Wing Virus has earlier been shown to be a contributory factor to winter mortality and Colony Collapse Disorder. Moreover, negative-strand detection of Sacbrood Virus in eggs was demonstrated for the first time.

**Conclusions:**

High pathogen loads were observed in this sanitary control program. We documented for the first time vertical transmission of some viruses, as well as significant differences between two honey bee races in being affected by Deformed Wing Virus. Nevertheless, we could not demonstrate a correlation between the presence of viruses and queen breeding efficacies.

**Electronic supplementary material:**

The online version of this article (doi:10.1186/s12917-015-0386-9) contains supplementary material, which is available to authorized users.

## Background

In view of the spread of varroasis – a mite infestation of the honey bee – over Europe and the problems which this disease has brought about in the beekeeping sector, the Member States of the European Union have been encouraged to set up national programmes aimed at improving the general conditions for the production and marketing of apicultural products. In Belgium, such apicultural programmes now exist for many years and particularly in the Flemish region, a lot of effort has been put in the restocking of hives. Within this programme, a limited number of recognized breeders are provided with the possibility to travel to a land mating yard in Belgium (Kreverhille) and island mating yards in Germany (Spiekeroog, Norderney) and the Netherlands (Ameland, Marken) with selected virgin queens. When these fertilized queens perform well they become the new breeding queens two years later, and are distributed on a large scale among the other beekeepers. Overall, this programme enjoyed a high participation rate amongst the beekeepers, but failed to a certain extent in terms of the efficacy of the queen breeding programme. This is evident from the fact that in the past four years, between 5,948 and 6,195 larvae were grafted, but only 61.4-70.8% could be raised to newborn queens and from these only 75.0-79.9% became egg-laying. Thus overall, the success ratio of the queen breeding programme has been only 49.1-53.1%, a fairly low number [1].

One of the measures that were taken to tackle this low breeding efficacy was the publication and distribution of a technical brochure describing the proper way to introduce a new queen into a bee colony. Since the problems persisted, we subsequently organized sanitary controls of the breeding queens in 2012 and 2014. This measure was taken given that honey bees can be exposed to several single stranded RNA viruses and transmission can occur both horizontally and vertically (reviewed by Chen *et al*. [2,3]). In horizontal transmission, viruses are transmitted among individuals of the same generation. Vertical transmission occurs from mothers to their offspring and can have two main causes: (I) infected sperm originating from the drones and (II) contaminated eggs originating from infected spermatheca and/or ovaries of the queen. The reproducing individuals, the queen and the drones, have a protective status in the colony because they are fed by the nurse bees. Nevertheless, both castes are susceptible to parasites. Several viruses were already demonstrated in individual queens and drones [4-9]. The presence of viruses in reproductive tissues of queens and drones were also investigated [10-14].

A non-destructive method to investigate whether vertical transmission occurs relies on examination of freshly laid eggs. In this study, we focused on a number of commonly occurring bee viruses [3] e.g. Deformed Wing Virus (DWV), but also on a set of viruses that were recently discovered in the USA such as Lake Sinai Virus (LSV) [15], and which we discovered to be present in Belgian apiaries as well [16]. Moreover, using the BeeDoctor diagnostic tool [17] which is based on the multiplex ligation-dependent probe amplification technology, we were also able to screen in parallel for the negative-strand intermediate.

Both *Apis mellifera carnica*-breeders and Buckfast-breeders participated in our study. *Apis mellifera carnica* or the carniolan honey bee is the subspecies of the European honey bee native to the Balkan Peninsula and represents the majority of Belgian bee populations due to massive import. This race is favoured for several reasons, e.g. non-aggressiveness and honey yield. The Buckfast bee is a combination race, a cross of various *Apis mellifera* subspecies and was developed in the United Kingdom during several decades.

## Methods

Flemish honey bee queen breeders were instructed to collect 10 eggs from worker cells from the same honey bee colony, per sample. In the summer of 2012, 35 queen breeders collected a sample from one colony each. In 2014, a further 43 egg samples were obtained. This set originated from 11 queen breeders, who surveyed each several colonies, varying from one to nine. This resulted in a total of 78 egg samples used in this study. The eggs were preserved at −20°C, transported to the laboratory on dry ice and then stored at −80°C until the RNA was isolated, using the RNeasy Lipid Tissue (Qiagen). The eggs were homogenised in the presence of zirconium beads and 0.5 ml QIAzol lysis reagent (Qiagen). Using random hexamer primers, 200 ng RNA was retro-transcribed with the RevertAid H Minus First Strand cDNA Synthesis Kit (Thermo Scientific).

The eggs were examined by RT PCR assays for the presence of viruses of the Acute Bee Paralysis Virus (ABPV) complex [18], Aphid Lethal Paralysis Virus strain Brookings (ALPV) [16], Black Queen Cell Virus (BQCV) [19], Chronic Bee Paralysis Virus (CBPV) [20], DWV [21], LSV [16], Sacbrood Virus (SBV) [19] and *Varroa destructor* Macula-like Virus (VdMLV) [11]. Samples positive for the ABPV complex were re-analysed with specific primers for ABPV [22], Israeli Acute Bee Paralysis Virus [23] and Kashmir Bee Virus [22]. We used honey bee β-actin [24] as a control gene to monitor the efficiency of the PCR reaction and its previous steps. All PCR reaction mixtures contained: 2 μM of each primer; 1 mM MgCl_2_; 0.2 mM dNTPs; 1.2 U Hotstar Taq DNA polymerase (Qiagen) and 2 μl cDNA product. Positive samples of each detected virus, except CBPV and VdMLV, were analysed for their negative-strand. This was detected with the BeeDoctor tool [17] in its uniplex modus, using 3 μl RNA.

PCR products were separated by electrophoresis using 1.4% agarose gels or 4% high resolution agarose gels for the MLPA PCR products, stained with ethidium bromide and visualised under UV light. Amplicons of each virus were sequenced on an ABI 3130XL platform with M13 primers after cloning with the TOPO TA Cloning Kit for sequencing (Invitrogen). DNA sequences were analysed using Geneious R7 to confirm the identity.

The incidence of the screened viruses (percentage infected) as well as the total virus load (total number of detected viruses) in *carnica* and Buckfast bees was compared using binomial and Poisson generalized linear mixed models with function glmer in package lme4 v. 1.1-7 in R v. 3.1.1. In these analyses, race and year were coded as fixed factors and breeder was coded as a random factor, and significance was assessed using Wald tests. Least square means on average infection percentages and total virus load and 95% Wald confidence limits were calculated using the effects package v. 3.0-3. Finally, a linear regression analysis was used to test the effect of virus load (total number of infecting viruses) on the percentage of queens that were born from grafted larvae, the percentage of queens that went on to lay out of all larvae that were grafted and the percentage of all queens that were born that went on to lay. This analysis was performed in GraphPad Prism 6.

## Results and discussion

**Figure 1:**
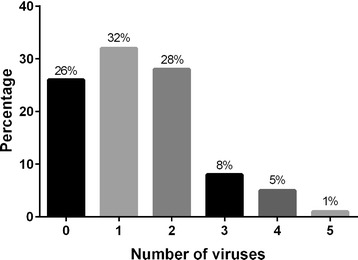
**Number of detected viruses and their prevalences.** The samples used in our study were co-infected with a number of viruses, ranging from 1 to 5. In almost 26% of the samples were no viruses detected.

In this study, we found a high prevalence of different honey bee viruses in eggs used in queen breeding operations (Additional file 1: Table S1). Although we investigated representative samples consisting of ten eggs per sample, false negatives can be present. Over two sampling years, 75% (58/78) of the egg samples were infected with at least one virus whereof 32% (25/78) of the samples were infected with a single virus and 42% (33/78) were infected with multiple viruses (Figure 1).

**Figure 2:**
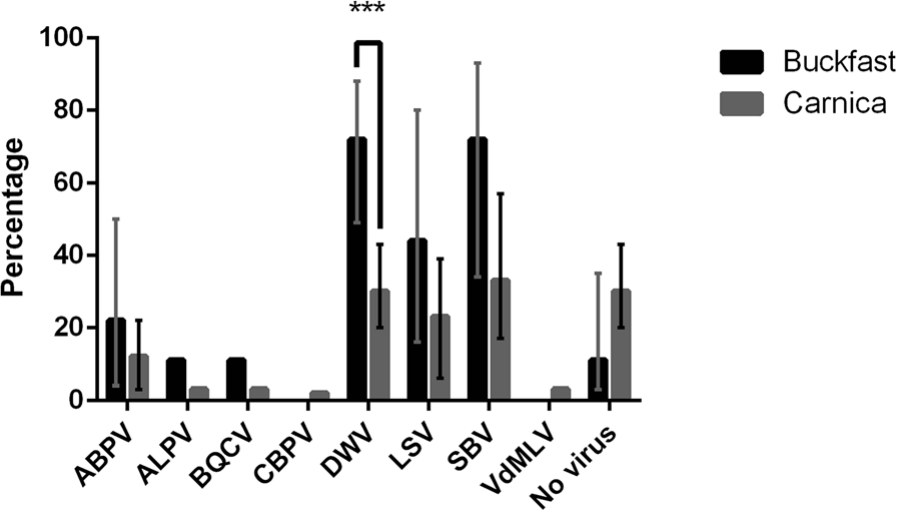
**Comparison of the incidence of different viruses in** ***Apis mellifera carnica*** **and Buckfast bees, together with 95% confidence limits based on fitted binomial mixed models (incidence over the sampling years 2012 and 2014 was averaged and bee breeder was included as a random factor,** ***n*** **= 78 samples).** Accurate confidence limits could not be calculated for species with very low infection rates (≤5%), and are omitted from this graph. ***: significant difference with *p* < 0.01 (Wald tests, binomial GLMM).

The most abundantly detected viruses were DWV (40%, 31/78) and SBV (42%, 33/78). LSV and ABPV were moderately detected in 28% (22/78) and 14% (11/78) of the samples. The other viruses ALPV, BQCV, CBPV and VdMLV had only low prevalences, respectively 5% (4/78), 5% (4/78), 1% (1/78) and 3% (2/78). Remarkably, *carnica* had a significantly lower infection rate with DWV than Buckfast [binomial GLMM, *z* = −3.048, *p* = 0.002, 30% mean infection rate in *carnica* ([20%, 43%] 95% C.L.) vs. 73% mean infection rate in Buckfast ([49%, 88%] 95% C.L.)] (Figure 2) as well as a significantly lower total virus load (total number of detected viruses) per sample [Poisson GLMM, *z* = −3.911, *p* = 9.10^−5^, average of 1.1 infecting viruses in *carnica* ([0.8, 1.4] 95% C.L.) vs. an average of 2.3 infecting viruses in Buckfast ([1.7, 3.2] 95% C.L.)]. No significant differences were found in the incidence of the other viruses screened (binomial GLMM, *p* > 0.05).

Our results, however, did not indicate a correlation between the virus burden (total number of infecting viruses) and queen breeding efficacy (Additional file 2: Figure S1). It might be the case though that variation in beekeeping management skills required for successful queen breeding [1] hides any effect of virus burden on queen breeding efficacy. Given the important effects that some of the viruses detected here have on honey bee health, including a large effect on winter mortality [25-28], delayed negative effects on honey bee health are likely, particularly given the implied vertical transmission to offspring workers. Indeed, this study is the first to document vertical transmission for ALPV, LSV and VdMLV. This is another confirmation that these viruses can infect honey bees, especially given that the negative strand was previously detected [15,16]. Moreover, BQCV lethally affects developing queen larvae and pupae. After death of the pupae, the wall of the queen cell eventually colours dark [3]. This virus is reported to be a common cause of queen larvae mortality [29] and is correlated with the queenless condition of an apiary [26].

Furthermore, we have detected the negative-strand of SBV. Although this might indicate that SBV replicates in eggs, it is also possible that this originates from transovum transmission, such as surface contamination with sperm containing negative-strand RNAs. Replication of SBV was previously reported in adults and larvae of European (*A. mellifera*) and Asian honey bees (*Apis cerana*) [30-32]. This virus is frequently found in adult bees that are covertly infected. A Belgian screening of adult forager bees revealed a prevalence of 19% [17], but this varies greatly in other European countries [22,33,34]. Larvae can be overtly infested, which then results in a failure to pupate and eventually death [3]. Nonetheless, problems with this virus are seldom reported by beekeepers, in contrast to the Asian serotypes that infect *A. cerana* [35,36]. Although SBV is mainly horizontally transmitted, its detection in eggs demonstrated that vertical transmission also occurs. It can be expected that a replicating virus in honey bee eggs can have consequences for the development into a queen, resulting in a clinical relevance for queen breeding, and can also have knock-on effects after being transmitted to the offspring workers or drones [3].

A broad virus screening of honey bee eggs was not yet performed. Nevertheless, few studies reported the presence of viruses [6-8,19,37] but only limited numbers of colonies were screened. However, our study of fertilised eggs does not allow us to pinpoint the infection source, queen or drone, which could be important for eventual remedial actions. Because surface-sterilisation was not applicable in our study design, we could not distinguish between viruses on the surface of the eggs (transovum transmission) or within the eggs (transovarian transmission). Because of the possible transovum transmission, the emerging larvae will not necessarily be infected with viruses as previously demonstrated [6]. Nevertheless, these larvae are exposed to horizontal transmission via feeding (reviewed by Chen *et al*. [2]).

## Conclusions

A survey of viruses in honey bee eggs in the context of a queen breeding program demonstrated high incidences of two viruses (DWV and SBV) and moderate to low incidences of a further six viruses (ABPV, ALPV, BQCV, CBPV, LSV and VdMLV). Vertical transmission (transovum or transovarian) of some viruses (ALPV, LSV, VdMLV) was demonstrated for the first time as well as negative-strand detection of SBV. We could not demonstrate a correlation between the presence of viruses and the low queen breeding efficacies. Remarkably, we found *Apis mellifera carnica* bees to be less infected with Deformed Wing Virus (p < 0.01) than Buckfast bees, and also found them to have a lower average total number of infecting viruses (p < 0.001). This is a significant finding, given that Deformed Wing Virus has earlier been shown to be a contributory factor to winter mortality, and offers interesting perspectives for breeding virus-resistant bees. However, we cannot make general conclusions about the virus-resistant state of *carnica* race compared to Buckfast race solely based on our data. Concluding, further sanitary screenings in the context of queen breeding seems advisory, especially because BQCV infection is a common cause of queen larval death [29].

### Ethics statement

The study involved the European honey bee (*Apis mellifera*), which is neither an endangered nor a protected species.

## Additional files

Additional file 1: Table S1. Overview of the detected viruses in honeybee egg samples, subdivided per year. For each sample is the corresponding apiary, bee race and total virus burden shown. The virus prevalence per sampling year and the overall occurrence are indicated. Additional file 2: Figure S1. Linear regression analysis of the effect of virus loads (total number of detected viruses) on queen breeding efficacies: effect on (A) the percentage of queens that were born from grafted larvae, (B) the percentage of queens that went on to lay out of all larvae that were grafted and (C) the percentage of all queens that were born that went on to lay.
