# Supplementary material for: Vertical transmission of honey bee viruses in a Belgian queen breeding program
Source: BMC Vet Res. 2015 Mar 14;11:61. doi: 10.1186/s12917-015-0386-9 (PMC4365526; doi:10.1186/s12917-015-0386-9)
Supplement: Additional file 1: Table S1. — Overview of the detected viruses in honeybee egg samples, subdivided per year. For each sample is the corresponding apiary, bee race and total virus burden shown. The virus prevalence per sampling year and the overall occurrence are indicated. [file 12917_2015_386_MOESM1_ESM.docx]

**Table S1**: Overview of the detected viruses in honeybee egg samples, subdivided per year. For each sample is the corresponding apiary, bee race and total virus burden shown. The virus prevalence per sampling year and the overall occurrence are indicated.

ABPV: Acute Bee Paralysis Virus, ALPV: Aphid Lethal Paralysis Virus strain Brookings, BQCV: Black Queen Cell Virus, CBPV: Chronic Bee Paralysis Virus, DWV: Deformed Wing Virus, LSV: Lake Sinai Virus, SBV: Sacbrood Virus and VdMLV: *Varroa destructor* Macula-like Virus

| **SAMPLING IN 2012** | | | | | | | | | | | |
| --- | --- | --- | --- | --- | --- | --- | --- | --- | --- | --- | --- |
| **Apiary** | **Race** | **ABPV** | **ALPV** | **BQCV** | **CBPV** | **DWV** | **LSV** | **SBV** | **VdMLV** | **Negative** | **Virus load** |
| 1 | Buckfast | 0 | 0 | 0 | 0 | 1 | 0 | 1 | 0 | 0 | 2 |
| 2 | Buckfast | 0 | 0 | 0 | 0 | 1 | 0 | 1 | 0 | 0 | 2 |
| 3 | Buckfast | 0 | 0 | 0 | 0 | 1 | 0 | 1 | 0 | 0 | 2 |
| 4 | Buckfast | 0 | 0 | 0 | 0 | 0 | 0 | 0 | 0 | 1 | 0 |
| 5 | Buckfast | 0 | 0 | 0 | 0 | 0 | 0 | 0 | 0 | 1 | 0 |
| 6 | Buckfast | 0 | 0 | 0 | 0 | 1 | 0 | 0 | 0 | 0 | 1 |
| 7 | Buckfast | 0 | 0 | 0 | 0 | 1 | 1 | 0 | 0 | 0 | 2 |
| 8 | Buckfast | 0 | 1 | 0 | 0 | 1 | 1 | 1 | 0 | 0 | 4 |
| 9 | Buckfast | 0 | 0 | 0 | 0 | 0 | 0 | 1 | 0 | 0 | 1 |
| 10 | Carnica | 0 | 0 | 0 | 0 | 0 | 0 | 1 | 0 | 0 | 1 |
| 11 | Carnica | 0 | 0 | 0 | 0 | 0 | 0 | 1 | 0 | 0 | 1 |
| 12 | Carnica | 0 | 0 | 0 | 0 | 0 | 0 | 1 | 0 | 0 | 1 |
| 13 | Carnica | 0 | 0 | 0 | 0 | 0 | 0 | 1 | 0 | 0 | 1 |
| 14 | Carnica | 0 | 0 | 0 | 0 | 1 | 0 | 1 | 0 | 0 | 2 |
| 15 | Carnica | 0 | 0 | 0 | 0 | 0 | 0 | 0 | 0 | 1 | 0 |
| 16 | Carnica | 0 | 0 | 0 | 0 | 0 | 0 | 0 | 0 | 1 | 0 |
| 17 | Carnica | 0 | 0 | 0 | 0 | 0 | 1 | 1 | 0 | 0 | 2 |
| 18 | Carnica | 0 | 0 | 0 | 0 | 0 | 0 | 0 | 0 | 1 | 0 |
| 19 | Carnica | 0 | 0 | 0 | 0 | 0 | 1 | 1 | 0 | 0 | 2 |
| 20 | Carnica | 0 | 0 | 0 | 0 | 0 | 0 | 0 | 0 | 1 | 0 |
| 21 | Carnica | 0 | 0 | 0 | 0 | 1 | 0 | 0 | 0 | 0 | 1 |
| 22 | Carnica | 0 | 0 | 0 | 0 | 0 | 0 | 0 | 0 | 1 | 0 |
| 23 | Carnica | 0 | 0 | 0 | 0 | 0 | 0 | 0 | 0 | 1 | 0 |
| 24 | Carnica | 1 | 0 | 0 | 0 | 0 | 0 | 1 | 0 | 0 | 2 |
| 25 | Carnica | 0 | 0 | 0 | 0 | 1 | 0 | 1 | 0 | 0 | 2 |
| 26 | Carnica | 0 | 0 | 0 | 0 | 0 | 0 | 1 | 0 | 0 | 1 |
| 27 | Carnica | 0 | 0 | 0 | 0 | 1 | 0 | 1 | 0 | 0 | 2 |
| 28 | Carnica | 0 | 1 | 0 | 0 | 1 | 0 | 0 | 0 | 0 | 2 |
| 29 | Carnica | 0 | 0 | 0 | 0 | 1 | 0 | 0 | 0 | 0 | 1 |
| 30 | Carnica | 0 | 0 | 0 | 0 | 0 | 1 | 0 | 0 | 0 | 1 |
| 31 | Carnica | 1 | 0 | 0 | 0 | 0 | 0 | 0 | 0 | 0 | 1 |
| 32 | Carnica | 0 | 0 | 0 | 0 | 0 | 0 | 0 | 0 | 1 | 0 |
| 33 | Carnica | 0 | 0 | 0 | 0 | 1 | 0 | 1 | 0 | 0 | 2 |
| 34 | Carnica | 0 | 0 | 0 | 0 | 0 | 0 | 1 | 0 | 0 | 1 |
| 35 | Carnica | 0 | 0 | 0 | 0 | 0 | 0 | 1 | 0 | 0 | 1 |
| **Total** | | 2 | 2 | 0 | 0 | 13 | 5 | 19 | 0 | 9 | 41 |
| **Percentage** | | 6% | 6% | 0,0% | 0,0% | 37% | 14% | 54% | 0,0% | 26% | N.A. |
| **SAMPLING IN 2014** | | | | | | | | | | | |
| **Apiary** | **Race** | **ABPV** | **ALPV** | **BQCV** | **CBPV** | **DWV** | **LSV** | **SBV** | **VdMLV** | **Negative** | **Virus load** |
| 1 | Buckfast | 1 | 0 | 0 | 0 | 1 | 1 | 1 | 0 | 0 | 4 |
| 1 | Buckfast | 0 | 0 | 0 | 0 | 1 | 1 | 1 | 0 | 0 | 3 |
| 1 | Buckfast | 1 | 0 | 1 | 0 | 1 | 1 | 1 | 0 | 0 | 5 |
| 1 | Buckfast | 1 | 0 | 0 | 0 | 1 | 0 | 1 | 0 | 0 | 3 |
| 1 | Buckfast | 0 | 0 | 0 | 0 | 1 | 0 | 1 | 0 | 0 | 2 |
| 2 | Buckfast | 1 | 0 | 0 | 0 | 0 | 1 | 0 | 0 | 0 | 2 |
| 2 | Buckfast | 0 | 1 | 0 | 0 | 0 | 0 | 1 | 0 | 0 | 2 |
| 2 | Buckfast | 0 | 0 | 1 | 0 | 1 | 1 | 1 | 0 | 0 | 4 |
| 3 | Buckfast | 0 | 0 | 0 | 0 | 1 | 1 | 1 | 0 | 0 | 3 |
| 4 | Carnica | 0 | 0 | 0 | 0 | 0 | 1 | 1 | 0 | 0 | 2 |
| 4 | Carnica | 0 | 0 | 0 | 0 | 0 | 1 | 1 | 0 | 0 | 2 |
| 5 | Carnica | 0 | 0 | 0 | 0 | 0 | 0 | 1 | 0 | 0 | 1 |
| 5 | Carnica | 1 | 0 | 0 | 0 | 1 | 0 | 1 | 0 | 0 | 3 |
| 6 | Carnica | 0 | 0 | 0 | 0 | 1 | 1 | 0 | 0 | 0 | 2 |
| 6 | Carnica | 1 | 0 | 0 | 0 | 0 | 1 | 0 | 0 | 0 | 2 |
| 6 | Carnica | 0 | 0 | 1 | 0 | 1 | 1 | 0 | 1 | 0 | 4 |
| 6 | Carnica | 0 | 0 | 0 | 0 | 0 | 0 | 0 | 0 | 1 | 0 |
| 6 | Carnica | 0 | 1 | 0 | 0 | 1 | 0 | 0 | 0 | 0 | 2 |
| 6 | Carnica | 0 | 0 | 0 | 0 | 0 | 0 | 0 | 0 | 1 | 0 |
| 7 | Carnica | 0 | 0 | 0 | 0 | 0 | 1 | 1 | 0 | 0 | 2 |
| 7 | Carnica | 0 | 0 | 0 | 0 | 1 | 1 | 0 | 1 | 0 | 3 |
| 8 | Carnica | 0 | 0 | 0 | 0 | 0 | 1 | 0 | 0 | 0 | 1 |
| 8 | Carnica | 0 | 0 | 0 | 0 | 0 | 0 | 0 | 0 | 1 | 0 |
| 8 | Carnica | 1 | 0 | 0 | 0 | 0 | 1 | 0 | 0 | 0 | 2 |
| 8 | Carnica | 0 | 0 | 0 | 0 | 0 | 1 | 0 | 0 | 0 | 1 |
| 9 | Carnica | 0 | 0 | 0 | 0 | 0 | 1 | 0 | 0 | 0 | 1 |
| 9 | Carnica | 0 | 0 | 0 | 0 | 1 | 0 | 0 | 0 | 0 | 1 |
| 9 | Carnica | 0 | 0 | 0 | 0 | 0 | 0 | 0 | 0 | 1 | 0 |
| 9 | Carnica | 1 | 0 | 0 | 0 | 0 | 0 | 0 | 0 | 0 | 1 |
| 9 | Carnica | 1 | 0 | 0 | 0 | 0 | 0 | 0 | 0 | 0 | 1 |
| 10 | Carnica | 0 | 0 | 0 | 0 | 1 | 0 | 0 | 0 | 0 | 1 |
| 10 | Carnica | 0 | 0 | 0 | 0 | 0 | 0 | 0 | 0 | 1 | 0 |
| 10 | Carnica | 0 | 0 | 1 | 0 | 1 | 0 | 1 | 0 | 0 | 3 |
| 10 | Carnica | 0 | 0 | 0 | 0 | 0 | 0 | 0 | 0 | 1 | 0 |
| 11 | Carnica | 0 | 0 | 0 | 1 | 0 | 0 | 0 | 0 | 0 | 1 |
| 11 | Carnica | 0 | 0 | 0 | 0 | 0 | 0 | 0 | 0 | 1 | 0 |
| 11 | Carnica | 0 | 0 | 0 | 0 | 0 | 0 | 0 | 0 | 1 | 0 |
| 11 | Carnica | 0 | 0 | 0 | 0 | 0 | 0 | 0 | 0 | 1 | 0 |
| 11 | Carnica | 0 | 0 | 0 | 0 | 1 | 0 | 0 | 0 | 0 | 1 |
| 11 | Carnica | 0 | 0 | 0 | 0 | 0 | 0 | 0 | 0 | 1 | 0 |
| 11 | Carnica | 0 | 0 | 0 | 0 | 1 | 0 | 0 | 0 | 0 | 1 |
| 11 | Carnica | 0 | 0 | 0 | 0 | 1 | 0 | 0 | 0 | 0 | 1 |
| 11 | Carnica | 0 | 0 | 0 | 0 | 0 | 0 | 0 | 0 | 1 | 0 |
| **Total** | | 9 | 2 | 4 | 1 | 18 | 17 | 14 | 2 | 11 | 67 |
| **Percentage** | | 21% | 5% | 9% | 2% | 42% | 40% | 33% | 5% | 26% | N.A. |
| **Overall prevalence** | | 14% | 5% | 5% | 1% | 40% | 28% | 42% | 3% | 26% | N.A. |
